# 1,1′-Methyl­enebis[3-(2,6-diiso­propyl­phen­yl)-3,4,5,6-tetra­hydro­pyrimidin-1-ium] dibromide ethanol monosolvate monohydrate

**DOI:** 10.1107/S1600536813021004

**Published:** 2013-08-03

**Authors:** Huanyu Bian, Liangru Yang, Jinwei Yuan, Pu Mao, Yongmei Xiao

**Affiliations:** a College of Chemistry and Chemical Engineering, Henan University of Technology, Zhengzhou 450001, People’s Republic of China

## Abstract

In the title methyl­ene-bridged di(tetra­hydro­pyrimidinium) salt, C_33_H_50_N_4_^2+^·2Br^−^·C_2_H_5_OH·H_2_O, the two tetra­hydro­pyrimidinium rings have envelope conformations with the central CH_2_ C atom as the flap. Their mean planes are inclined to one another by 73.31 (13)° and the attached benzene rings are inclined to one another by 67.39 (15)°. The methylene-C—N bond lengths in the tetra­hydro­pyrimidinium rings are 1.314 (3) and 1.304 (3) Å, values typical for C=N double bonds. The distances between the methyl­ene-bridge C atom and the linked tetra­hydro­pyrimidinium N atom are 1.457 (3) and 1.465 (3) Å, values typical for C—N single bonds. The mol­ecules co-crystallized with H_2_O and EtOH mol­ecules from the solvent. In the crystal, there is a zigzag chain along [010] of water mol­ecules linked by one of the Br^−^ anions *via* O—H⋯Br hydrogen bonds. The second Br^−^ anion is hydrogen bonded (O—H⋯Br) to the ethanol solvent mol­ecule. There are also a number of C—H⋯Br and C—H⋯O hydrogen bonds present, leading to the formation of a two-dimensional network lying parallel to the *bc* plane.

## Related literature

For the synthesis of the precursor bis­(3-(2,6-diisopropyl-phen­yl)-hexa­hydro­pyrimidin­yl)methane, see: Bisceglia *et al.* (2004 ▶). For metal complexes of substituted 1,4,5,6-tetra­hydro­pyrimidines, see: Mao *et al.* (2012 ▶).
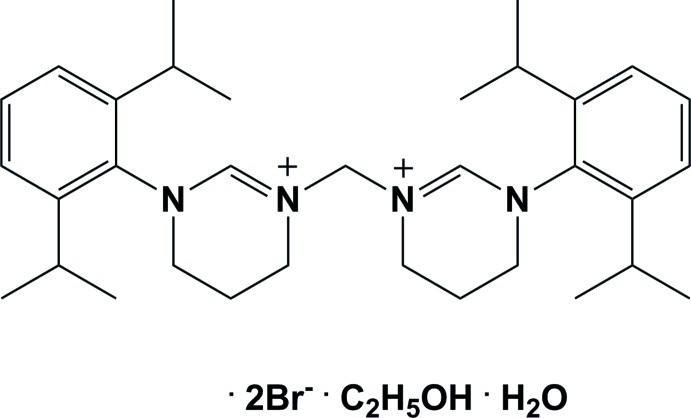


## Experimental

### 

#### Crystal data

C_33_H_50_N_4_^2+^·2Br^−^·C_2_H_6_O·H_2_O
*M* *_r_* = 726.67
Monoclinic, 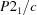

*a* = 13.6267 (4) Å
*b* = 10.3769 (2) Å
*c* = 26.9387 (6) Å
β = 91.361 (2)°
*V* = 3808.13 (16) Å^3^
*Z* = 4
Mo *K*α radiation
μ = 2.16 mm^−1^
*T* = 291 K
0.40 × 0.35 × 0.30 mm

#### Data collection

Agilent Xcalibur (Eos, Gemini) diffractometer
Absorption correction: multi-scan (*CrysAlis PRO*; Agilent, 2011 ▶) *T*_min_ = 0.825, *T*_max_ = 1.000
20153 measured reflections
7763 independent reflections
5411 reflections with *I* > 2σ(*I*)
*R*_int_ = 0.037

#### Refinement

*R*[*F* ^2^ > 2σ(*F* ^2^)] = 0.047
*wR*(*F* ^2^) = 0.100
*S* = 1.03
7763 reflections
406 parameters
2 restraints
H atoms treated by a mixture of independent and constrained refinement
Δρ_max_ = 0.53 e Å^−3^
Δρ_min_ = −0.40 e Å^−3^

### 

Data collection: *CrysAlis PRO* (Agilent, 2011 ▶); cell refinement: *CrysAlis PRO*; data reduction: *CrysAlis PRO*; program(s) used to solve structure: *SHELXS97* (Sheldrick, 2008 ▶); program(s) used to refine structure: *SHELXL97* (Sheldrick, 2008 ▶); molecular graphics: *OLEX2* (Dolomanov *et al.*, 2009 ▶); software used to prepare material for publication: *OLEX2*.

## Supplementary Material

Crystal structure: contains datablock(s) I, global. DOI: 10.1107/S1600536813021004/su2619sup1.cif

Structure factors: contains datablock(s) I. DOI: 10.1107/S1600536813021004/su2619Isup2.hkl

Supplementary material file. DOI: 10.1107/S1600536813021004/su2619Isup3.cml

Additional supplementary materials:  crystallographic information; 3D view; checkCIF report

## Figures and Tables

**Table 1:** Hydrogen-bond geometry (Å, °)

*D*—H⋯*A*	*D*—H	H⋯*A*	*D*⋯*A*	*D*—H⋯*A*
O2—H2*A*⋯Br1	0.92 (2)	2.41 (3)	3.324 (4)	172 (10)
O2—H2*B*⋯Br1^i^	0.93 (2)	2.42 (2)	3.339 (4)	170 (5)
O1—H1⋯Br2	0.82	2.45	3.262 (3)	171
C3—H3*B*⋯Br1	0.97	2.83	3.660 (3)	144
C4—H4⋯Br2^ii^	0.93	2.85	3.733 (2)	158
C5—H5*A*⋯O1	0.97	2.50	3.383 (4)	151
C5—H5*B*⋯Br2^ii^	0.97	2.73	3.670 (3)	163
C6—H6*B*⋯Br1	0.97	2.89	3.741 (3)	147
C9—H9⋯O1	0.93	2.30	3.197 (4)	161

Symmetry codes: (i) 
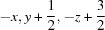
; (ii) 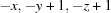.

**Fig. 1.**
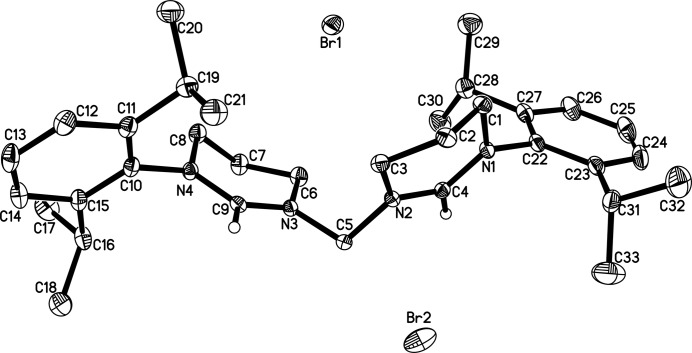
The molecular structure of the title molecule, with atom labelling. The displacement ellipsoids are drawn at the 30% probability level. Hydrogen atoms and solvent molecules have been omitted for clarity.

## supplementary crystallographic information

### 1. Comment

Our group is interested in the development of new *N*-heterocyclic carbene (NHC) ligands based on substituted 1,4,5,6-tetrahydropyrimidine, and their metal complexes (Mao *et al.*, 2012). In the course of the metallation of methylene bridged tetrahydropyrimidinium salts, we observed that the methylene linkage broke during the metallation process. In the search for possible reasons leading to the breakage of the linkage, we carried out the X-ray crystal structure analysis of the methylene bridged tetrahydropyrimidinium dibromide.

In the structure of the title compound, Fig. 1, the C—N of the tetrahydropyrimidinium ring and the linkage showed typical values of double and single bonds, respectively. The title molecule co-crystallized with H_2_O and EtOH molecules from the solvent. The two tetrahydropyrimidinium rings have envelope conformations with the central CH_2_ C atoms, C2 and C7, as the flaps. Their mean planes are inclined to one another by 73.31 (13) ° and the attached benzene rings are inclined to one another by 67.39 (15) °.

In the crystal, there is a zigzag chain along [010] of water molecules linked to one of the Br^-^ anions via O—H···Br hydrogen bonds (Table 1). The second Br^-^ anion is hydrogen bonded (O—H···Br) to the ethanol solvent molecule (Table 1). There are also a number of C—H···Br and C—H···O hydrogen bonds present, leading to the formation of a two-dimensional network lying parallel to the bc plane (Table 1).

### 2. Experimental

The starting product bis(3-(2,6-diisopropyl-phenyl)-hexahydropyrimidinyl)methane, was prepared by reaction of 1-(2,6-diisopropyl-phenyl)-propyl-1,3-diamine with aqueous formaldehyde in methanol, following a literature report (Bisceglia *et al.*, 2004). Bis(3-(2,6-diisopropyl-phenyl)-hexahydropyrimidinyl)methane (1.00 g, 1.98 mmmol) was then dissolved in absolute 1,2-dimethoxy-ethane (50 mL) and treated with *N*-bromosuccinimide (0.705 g, 3.96 mmol). The reaction mixture was stirred at room temperature for 2 h before the volatile compounds were removed in vacuo and a brown, oily residue remained. Crystallization of the residue in EtOH afforded colourless crystals of the title compound.

### 3. Refinement

The water H-atoms were located in a difference electron-density map and freely refined. The OH and C-bound H-atoms were included in calculated positions and treated as riding atoms: O-H = 0.82 Å, C-H = 0.93, 0.97 and 0.96 Å for CH, CH_2_, and CH_3_ H-atoms, respectively, with U_iso_(H) = k × U_eq_(C), where k = 1.5 for OH and CH_3_ H-atoms, and = 1.2 for other H-atoms.

### Crystal data

**Table:**

C_33_H_50_N_4_^2+^·2Br^−^·C_2_H_6_O·H_2_O	*F*(000) = 1528
*M**_r_* = 726.67	*D*_x_ = 1.267 Mg m^−^^3^
Monoclinic, *P*2_1_/*c*	Mo *K*α radiation, λ = 0.7107 Å
*a* = 13.6267 (4) Å	Cell parameters from 5778 reflections
*b* = 10.3769 (2) Å	θ = 3.0–26.3°
*c* = 26.9387 (6) Å	µ = 2.16 mm^−^^1^
β = 91.361 (2)°	*T* = 291 K
*V* = 3808.13 (16) Å^3^	Prismatic, colourless
*Z* = 4	0.40 × 0.35 × 0.30 mm

### Data collection

**Table:**

Agilent Xcalibur (Eos, Gemini) diffractometer	7763 independent reflections
Radiation source: Enhance (Mo) X-ray Source	5411 reflections with *I* > 2σ(*I*)
Graphite monochromator	*R*_int_ = 0.037
Detector resolution: 16.2312 pixels mm^-1^	θ_max_ = 26.4°, θ_min_ = 3.0°
ω scans	*h* = −17→16
Absorption correction: multi-scan (*CrysAlis PRO*; Agilent, 2011)	*k* = −11→12
*T*_min_ = 0.825, *T*_max_ = 1.000	*l* = −33→33
20153 measured reflections	

### Refinement

**Table:**

Refinement on *F*^2^	Primary atom site location: structure-invariant direct methods
Least-squares matrix: full	Secondary atom site location: difference Fourier map
*R*[*F*^2^ > 2σ(*F*^2^)] = 0.047	Hydrogen site location: inferred from neighbouring sites
*wR*(*F*^2^) = 0.100	H atoms treated by a mixture of independent and constrained refinement
*S* = 1.03	*w* = 1/[σ^2^(*F*_o_^2^) + (0.0353*P*)^2^ + 1.5781*P*] where *P* = (*F*_o_^2^ + 2*F*_c_^2^)/3
7763 reflections	(Δ/σ)_max_ = 0.001
406 parameters	Δρ_max_ = 0.53 e Å^−^^3^
2 restraints	Δρ_min_ = −0.40 e Å^−^^3^

### Special details

**Table:**

Geometry. All e.s.d.'s (except the e.s.d. in the dihedral angle between two l.s. planes) are estimated using the full covariance matrix. The cell e.s.d.'s are taken into account individually in the estimation of e.s.d.'s in distances, angles and torsion angles; correlations between e.s.d.'s in cell parameters are only used when they are defined by crystal symmetry. An approximate (isotropic) treatment of cell e.s.d.'s is used for estimating e.s.d.'s involving l.s. planes.
Refinement. Refinement of *F*^2^ against ALL reflections. The weighted *R*-factor *wR* and goodness of fit *S* are based on *F*^2^, conventional *R*-factors *R* are based on *F*, with *F* set to zero for negative *F*^2^. The threshold expression of *F*^2^ > σ(*F*^2^) is used only for calculating *R*-factors(gt) *etc*. and is not relevant to the choice of reflections for refinement. *R*-factors based on *F*^2^ are statistically about twice as large as those based on *F*, and *R*- factors based on ALL data will be even larger.

### Fractional atomic coordinates and isotropic or equivalent isotropic displacement parameters (Å^2^)

**Table:**

	*x*	*y*	*z*	*U*_iso_*/*U*_eq_	
Br1	−0.00021 (3)	0.39577 (3)	0.739687 (12)	0.05244 (12)	
Br2	0.15358 (3)	0.69268 (4)	0.506573 (13)	0.06818 (14)	
O1	0.3004 (2)	0.4779 (3)	0.55638 (12)	0.0927 (9)	
H1	0.2678	0.5316	0.5409	0.139*	
O2	0.1471 (3)	0.6392 (4)	0.77111 (16)	0.1121 (12)	
N1	−0.11949 (16)	0.62173 (19)	0.62054 (8)	0.0300 (5)	
N2	0.01884 (16)	0.50715 (19)	0.59859 (7)	0.0278 (5)	
N3	0.08481 (16)	0.29232 (19)	0.60165 (8)	0.0288 (5)	
N4	0.20292 (16)	0.17986 (19)	0.64720 (8)	0.0314 (5)	
C1	−0.0641 (2)	0.6922 (3)	0.65980 (10)	0.0350 (7)	
H1A	−0.0663	0.6448	0.6908	0.042*	
H1B	−0.0936	0.7762	0.6649	0.042*	
C2	0.0408 (2)	0.7081 (2)	0.64459 (10)	0.0371 (7)	
H2C	0.0786	0.7485	0.6713	0.045*	
H2D	0.0434	0.7636	0.6157	0.045*	
C3	0.0852 (2)	0.5776 (2)	0.63261 (10)	0.0340 (6)	
H3A	0.1483	0.5895	0.6174	0.041*	
H3B	0.0954	0.5286	0.6629	0.041*	
C4	−0.0745 (2)	0.5337 (2)	0.59437 (9)	0.0297 (6)	
H4	−0.1119	0.4872	0.5713	0.036*	
C5	0.0609 (2)	0.4029 (2)	0.56977 (9)	0.0313 (6)	
H5A	0.1199	0.4330	0.5540	0.038*	
H5B	0.0144	0.3766	0.5439	0.038*	
C6	0.0022 (2)	0.2149 (3)	0.61961 (11)	0.0388 (7)	
H6A	−0.0466	0.2039	0.5932	0.047*	
H6B	−0.0284	0.2590	0.6470	0.047*	
C7	0.0398 (2)	0.0843 (3)	0.63669 (12)	0.0422 (7)	
H7A	−0.0118	0.0384	0.6534	0.051*	
H7B	0.0588	0.0338	0.6082	0.051*	
C8	0.1273 (2)	0.1014 (3)	0.67170 (11)	0.0404 (7)	
H8A	0.1070	0.1438	0.7018	0.049*	
H8B	0.1543	0.0178	0.6806	0.049*	
C9	0.1756 (2)	0.2702 (2)	0.61571 (9)	0.0296 (6)	
H9	0.2242	0.3220	0.6025	0.036*	
C10	0.3050 (2)	0.1641 (3)	0.66192 (10)	0.0347 (7)	
C11	0.3474 (2)	0.2525 (3)	0.69526 (11)	0.0392 (7)	
C12	0.4457 (2)	0.2335 (3)	0.70865 (12)	0.0499 (8)	
H12	0.4765	0.2908	0.7305	0.060*	
C13	0.4980 (3)	0.1318 (3)	0.69028 (13)	0.0566 (9)	
H13	0.5637	0.1215	0.6996	0.068*	
C14	0.4540 (2)	0.0454 (3)	0.65826 (13)	0.0523 (8)	
H14	0.4903	−0.0237	0.6465	0.063*	
C15	0.3567 (2)	0.0587 (3)	0.64302 (11)	0.0408 (7)	
C16	0.3111 (2)	−0.0400 (3)	0.60767 (12)	0.0514 (8)	
H16	0.2449	−0.0105	0.5984	0.062*	
C17	0.3022 (3)	−0.1712 (3)	0.63334 (15)	0.0695 (11)	
H17A	0.2725	−0.2320	0.6107	0.104*	
H17B	0.2622	−0.1627	0.6620	0.104*	
H17C	0.3663	−0.2012	0.6434	0.104*	
C18	0.3695 (3)	−0.0539 (4)	0.56023 (13)	0.0683 (10)	
H18A	0.4334	−0.0879	0.5683	0.103*	
H18B	0.3760	0.0289	0.5448	0.103*	
H18C	0.3356	−0.1115	0.5378	0.103*	
C19	0.2922 (2)	0.3655 (3)	0.71713 (11)	0.0430 (7)	
H19	0.2232	0.3582	0.7064	0.052*	
C20	0.2963 (3)	0.3628 (3)	0.77382 (12)	0.0651 (10)	
H20A	0.2691	0.2831	0.7853	0.098*	
H20B	0.2591	0.4336	0.7865	0.098*	
H20C	0.3633	0.3699	0.7853	0.098*	
C21	0.3311 (3)	0.4933 (3)	0.69788 (13)	0.0615 (10)	
H21A	0.3985	0.5033	0.7083	0.092*	
H21B	0.2930	0.5628	0.7109	0.092*	
H21C	0.3263	0.4943	0.6623	0.092*	
C22	−0.2241 (2)	0.6421 (3)	0.61480 (10)	0.0348 (7)	
C23	−0.2563 (2)	0.7541 (3)	0.59017 (11)	0.0419 (7)	
C24	−0.3565 (3)	0.7721 (3)	0.58588 (13)	0.0589 (9)	
H24	−0.3806	0.8453	0.5698	0.071*	
C25	−0.4211 (3)	0.6851 (4)	0.60466 (15)	0.0653 (10)	
H25	−0.4883	0.6989	0.6006	0.078*	
C26	−0.3876 (2)	0.5773 (3)	0.62942 (13)	0.0566 (9)	
H26	−0.4326	0.5195	0.6422	0.068*	
C27	−0.2878 (2)	0.5531 (3)	0.63568 (11)	0.0413 (7)	
C28	−0.2532 (2)	0.4340 (3)	0.66426 (11)	0.0464 (8)	
H28	−0.1815	0.4383	0.6678	0.056*	
C29	−0.2952 (3)	0.4302 (4)	0.71605 (13)	0.0721 (11)	
H29A	−0.3654	0.4246	0.7135	0.108*	
H29B	−0.2768	0.5072	0.7337	0.108*	
H29C	−0.2698	0.3564	0.7336	0.108*	
C30	−0.2795 (3)	0.3113 (3)	0.63574 (14)	0.0677 (11)	
H30A	−0.3495	0.3055	0.6314	0.101*	
H30B	−0.2559	0.2377	0.6540	0.101*	
H30C	−0.2498	0.3133	0.6038	0.101*	
C31	−0.1854 (3)	0.8498 (3)	0.56818 (12)	0.0502 (8)	
H31	−0.1210	0.8351	0.5841	0.060*	
C32	−0.2133 (3)	0.9887 (3)	0.57786 (16)	0.0871 (14)	
H32A	−0.1667	1.0449	0.5627	0.131*	
H32B	−0.2133	1.0040	0.6130	0.131*	
H32C	−0.2776	1.0052	0.5640	0.131*	
C33	−0.1749 (4)	0.8245 (4)	0.51276 (15)	0.0951 (15)	
H33A	−0.1561	0.7364	0.5077	0.143*	
H33B	−0.1256	0.8805	0.4998	0.143*	
H33C	−0.2365	0.8407	0.4958	0.143*	
C34	0.3869 (3)	0.4560 (5)	0.53238 (17)	0.0906 (14)	
H34A	0.4293	0.5306	0.5364	0.109*	
H34B	0.3730	0.4445	0.4972	0.109*	
C35	0.4385 (4)	0.3408 (5)	0.55190 (18)	0.1017 (16)	
H35A	0.4931	0.3214	0.5313	0.153*	
H35B	0.3941	0.2690	0.5518	0.153*	
H35C	0.4617	0.3572	0.5852	0.153*	
H2A	0.112 (7)	0.567 (7)	0.761 (4)	0.38 (7)*	
H2B	0.105 (4)	0.709 (4)	0.772 (2)	0.16 (2)*	

### Atomic displacement parameters (Å^2^)

**Table:**

	*U*^11^	*U*^22^	*U*^33^	*U*^12^	*U*^13^	*U*^23^
Br1	0.0687 (3)	0.04436 (19)	0.04453 (19)	0.00286 (16)	0.00722 (16)	0.00699 (14)
Br2	0.0827 (3)	0.0748 (3)	0.0463 (2)	−0.0282 (2)	−0.01361 (19)	0.01094 (17)
O1	0.074 (2)	0.109 (2)	0.096 (2)	0.0035 (17)	0.0389 (18)	0.0350 (17)
O2	0.092 (3)	0.087 (2)	0.158 (3)	0.014 (2)	−0.008 (2)	−0.031 (2)
N1	0.0276 (13)	0.0288 (12)	0.0336 (12)	0.0041 (10)	0.0011 (10)	−0.0034 (9)
N2	0.0264 (13)	0.0276 (11)	0.0296 (11)	0.0009 (10)	0.0028 (9)	−0.0006 (9)
N3	0.0259 (13)	0.0287 (11)	0.0319 (12)	0.0018 (10)	0.0015 (10)	0.0008 (9)
N4	0.0288 (14)	0.0286 (12)	0.0367 (13)	0.0006 (10)	0.0018 (10)	0.0047 (10)
C1	0.0376 (18)	0.0340 (15)	0.0333 (15)	−0.0006 (13)	0.0009 (12)	−0.0060 (12)
C2	0.0390 (18)	0.0334 (15)	0.0390 (16)	−0.0062 (13)	0.0005 (13)	−0.0070 (12)
C3	0.0285 (16)	0.0408 (16)	0.0326 (14)	−0.0026 (13)	−0.0007 (12)	0.0011 (12)
C4	0.0321 (17)	0.0278 (14)	0.0292 (14)	−0.0001 (13)	−0.0003 (12)	0.0019 (11)
C5	0.0342 (16)	0.0327 (14)	0.0270 (14)	0.0053 (13)	0.0038 (12)	0.0007 (11)
C6	0.0272 (17)	0.0403 (16)	0.0488 (18)	−0.0052 (13)	0.0017 (13)	0.0033 (13)
C7	0.0360 (18)	0.0333 (16)	0.0574 (19)	−0.0053 (14)	0.0015 (15)	0.0080 (13)
C8	0.0361 (18)	0.0373 (15)	0.0480 (17)	−0.0010 (14)	0.0045 (14)	0.0119 (13)
C9	0.0303 (17)	0.0264 (14)	0.0325 (14)	0.0001 (12)	0.0069 (12)	−0.0022 (11)
C10	0.0275 (16)	0.0352 (15)	0.0414 (16)	−0.0001 (13)	−0.0019 (13)	0.0093 (12)
C11	0.0348 (18)	0.0391 (16)	0.0437 (17)	−0.0030 (14)	−0.0010 (14)	0.0094 (13)
C12	0.043 (2)	0.0499 (19)	0.057 (2)	−0.0084 (17)	−0.0092 (16)	0.0024 (15)
C13	0.0298 (19)	0.060 (2)	0.079 (3)	0.0037 (17)	−0.0086 (17)	0.0130 (18)
C14	0.036 (2)	0.0485 (19)	0.072 (2)	0.0095 (16)	−0.0011 (17)	0.0019 (17)
C15	0.0340 (18)	0.0368 (16)	0.0517 (18)	0.0053 (14)	0.0012 (14)	0.0052 (13)
C16	0.040 (2)	0.0454 (18)	0.069 (2)	0.0103 (16)	−0.0023 (17)	−0.0065 (16)
C17	0.070 (3)	0.050 (2)	0.089 (3)	−0.0072 (19)	0.010 (2)	−0.0083 (19)
C18	0.074 (3)	0.066 (2)	0.065 (2)	0.013 (2)	0.004 (2)	−0.0034 (19)
C19	0.0421 (19)	0.0441 (17)	0.0426 (18)	−0.0052 (15)	−0.0012 (14)	−0.0012 (13)
C20	0.080 (3)	0.067 (2)	0.049 (2)	0.003 (2)	0.0071 (19)	0.0005 (17)
C21	0.077 (3)	0.0452 (19)	0.062 (2)	−0.0038 (19)	0.0043 (19)	0.0010 (16)
C22	0.0320 (17)	0.0333 (15)	0.0389 (16)	0.0067 (13)	−0.0016 (13)	−0.0103 (12)
C23	0.045 (2)	0.0382 (16)	0.0427 (17)	0.0098 (15)	−0.0017 (14)	−0.0107 (13)
C24	0.051 (2)	0.052 (2)	0.073 (2)	0.0231 (19)	−0.0071 (19)	−0.0043 (17)
C25	0.031 (2)	0.073 (3)	0.092 (3)	0.0170 (19)	−0.0035 (19)	−0.014 (2)
C26	0.034 (2)	0.057 (2)	0.079 (2)	0.0004 (17)	0.0082 (17)	−0.0083 (18)
C27	0.0316 (18)	0.0418 (16)	0.0505 (18)	0.0033 (14)	0.0016 (14)	−0.0108 (14)
C28	0.0377 (19)	0.0449 (17)	0.057 (2)	−0.0037 (15)	0.0092 (15)	0.0022 (15)
C29	0.075 (3)	0.077 (3)	0.065 (2)	−0.002 (2)	0.012 (2)	0.0046 (19)
C30	0.078 (3)	0.049 (2)	0.077 (3)	0.0000 (19)	0.012 (2)	−0.0043 (18)
C31	0.059 (2)	0.0399 (17)	0.052 (2)	0.0096 (16)	−0.0053 (17)	0.0034 (14)
C32	0.116 (4)	0.045 (2)	0.101 (3)	0.000 (2)	0.001 (3)	−0.002 (2)
C33	0.134 (5)	0.091 (3)	0.062 (3)	−0.013 (3)	0.027 (3)	−0.007 (2)
C34	0.078 (3)	0.099 (3)	0.096 (3)	−0.014 (3)	0.030 (3)	0.014 (3)
C35	0.080 (4)	0.109 (4)	0.117 (4)	−0.003 (3)	0.024 (3)	0.016 (3)

### Geometric parameters (Å, º)

**Table:**

O1—H1	0.8200	C17—H17A	0.9600
O1—C34	1.376 (5)	C17—H17B	0.9600
O2—H2A	0.92 (2)	C17—H17C	0.9600
O2—H2B	0.928 (19)	C18—H18A	0.9600
N1—C1	1.478 (3)	C18—H18B	0.9600
N1—C4	1.314 (3)	C18—H18C	0.9600
N1—C22	1.446 (3)	C19—H19	0.9800
N2—C3	1.467 (3)	C19—C20	1.527 (4)
N2—C4	1.304 (3)	C19—C21	1.523 (4)
N2—C5	1.457 (3)	C20—H20A	0.9600
N3—C5	1.465 (3)	C20—H20B	0.9600
N3—C6	1.474 (3)	C20—H20C	0.9600
N3—C9	1.305 (3)	C21—H21A	0.9600
N4—C8	1.481 (3)	C21—H21B	0.9600
N4—C9	1.312 (3)	C21—H21C	0.9600
N4—C10	1.446 (3)	C22—C23	1.403 (4)
C1—H1A	0.9700	C22—C27	1.395 (4)
C1—H1B	0.9700	C23—C24	1.381 (4)
C1—C2	1.506 (4)	C23—C31	1.516 (4)
C2—H2C	0.9700	C24—H24	0.9300
C2—H2D	0.9700	C24—C25	1.367 (5)
C2—C3	1.521 (4)	C25—H25	0.9300
C3—H3A	0.9700	C25—C26	1.374 (5)
C3—H3B	0.9700	C26—H26	0.9300
C4—H4	0.9300	C26—C27	1.389 (4)
C5—H5A	0.9700	C27—C28	1.525 (4)
C5—H5B	0.9700	C28—H28	0.9800
C6—H6A	0.9700	C28—C29	1.521 (4)
C6—H6B	0.9700	C28—C30	1.526 (4)
C6—C7	1.517 (4)	C29—H29A	0.9600
C7—H7A	0.9700	C29—H29B	0.9600
C7—H7B	0.9700	C29—H29C	0.9600
C7—C8	1.512 (4)	C30—H30A	0.9600
C8—H8A	0.9700	C30—H30B	0.9600
C8—H8B	0.9700	C30—H30C	0.9600
C9—H9	0.9300	C31—H31	0.9800
C10—C11	1.399 (4)	C31—C32	1.515 (4)
C10—C15	1.404 (4)	C31—C33	1.526 (5)
C11—C12	1.393 (4)	C32—H32A	0.9600
C11—C19	1.519 (4)	C32—H32B	0.9600
C12—H12	0.9300	C32—H32C	0.9600
C12—C13	1.372 (4)	C33—H33A	0.9600
C13—H13	0.9300	C33—H33B	0.9600
C13—C14	1.373 (5)	C33—H33C	0.9600
C14—H14	0.9300	C34—H34A	0.9700
C14—C15	1.385 (4)	C34—H34B	0.9700
C15—C16	1.521 (4)	C34—C35	1.477 (6)
C16—H16	0.9800	C35—H35A	0.9600
C16—C17	1.534 (4)	C35—H35B	0.9600
C16—C18	1.528 (4)	C35—H35C	0.9600
			
C34—O1—H1	109.5	H17B—C17—H17C	109.5
H2A—O2—H2B	109 (8)	C16—C18—H18A	109.5
C4—N1—C1	119.4 (2)	C16—C18—H18B	109.5
C4—N1—C22	121.0 (2)	C16—C18—H18C	109.5
C22—N1—C1	119.3 (2)	H18A—C18—H18B	109.5
C4—N2—C3	122.3 (2)	H18A—C18—H18C	109.5
C4—N2—C5	120.4 (2)	H18B—C18—H18C	109.5
C5—N2—C3	117.3 (2)	C11—C19—H19	107.8
C5—N3—C6	117.3 (2)	C11—C19—C20	111.5 (3)
C9—N3—C5	120.3 (2)	C11—C19—C21	111.1 (3)
C9—N3—C6	122.3 (2)	C20—C19—H19	107.8
C9—N4—C8	119.4 (2)	C21—C19—H19	107.8
C9—N4—C10	120.9 (2)	C21—C19—C20	110.6 (3)
C10—N4—C8	119.4 (2)	C19—C20—H20A	109.5
N1—C1—H1A	109.8	C19—C20—H20B	109.5
N1—C1—H1B	109.8	C19—C20—H20C	109.5
N1—C1—C2	109.3 (2)	H20A—C20—H20B	109.5
H1A—C1—H1B	108.3	H20A—C20—H20C	109.5
C2—C1—H1A	109.8	H20B—C20—H20C	109.5
C2—C1—H1B	109.8	C19—C21—H21A	109.5
C1—C2—H2C	109.6	C19—C21—H21B	109.5
C1—C2—H2D	109.6	C19—C21—H21C	109.5
C1—C2—C3	110.2 (2)	H21A—C21—H21B	109.5
H2C—C2—H2D	108.1	H21A—C21—H21C	109.5
C3—C2—H2C	109.6	H21B—C21—H21C	109.5
C3—C2—H2D	109.6	C23—C22—N1	117.9 (3)
N2—C3—C2	109.5 (2)	C27—C22—N1	118.8 (2)
N2—C3—H3A	109.8	C27—C22—C23	123.3 (3)
N2—C3—H3B	109.8	C22—C23—C31	122.2 (3)
C2—C3—H3A	109.8	C24—C23—C22	116.7 (3)
C2—C3—H3B	109.8	C24—C23—C31	121.1 (3)
H3A—C3—H3B	108.2	C23—C24—H24	119.2
N1—C4—H4	117.7	C25—C24—C23	121.6 (3)
N2—C4—N1	124.5 (3)	C25—C24—H24	119.2
N2—C4—H4	117.7	C24—C25—H25	119.7
N2—C5—N3	110.73 (19)	C24—C25—C26	120.5 (3)
N2—C5—H5A	109.5	C26—C25—H25	119.7
N2—C5—H5B	109.5	C25—C26—H26	119.4
N3—C5—H5A	109.5	C25—C26—C27	121.3 (3)
N3—C5—H5B	109.5	C27—C26—H26	119.4
H5A—C5—H5B	108.1	C22—C27—C28	123.5 (3)
N3—C6—H6A	109.8	C26—C27—C22	116.6 (3)
N3—C6—H6B	109.8	C26—C27—C28	119.9 (3)
N3—C6—C7	109.3 (2)	C27—C28—H28	107.9
H6A—C6—H6B	108.3	C27—C28—C30	110.9 (3)
C7—C6—H6A	109.8	C29—C28—C27	111.4 (3)
C7—C6—H6B	109.8	C29—C28—H28	107.9
C6—C7—H7A	109.7	C29—C28—C30	110.6 (3)
C6—C7—H7B	109.7	C30—C28—H28	107.9
H7A—C7—H7B	108.2	C28—C29—H29A	109.5
C8—C7—C6	109.9 (2)	C28—C29—H29B	109.5
C8—C7—H7A	109.7	C28—C29—H29C	109.5
C8—C7—H7B	109.7	H29A—C29—H29B	109.5
N4—C8—C7	109.4 (2)	H29A—C29—H29C	109.5
N4—C8—H8A	109.8	H29B—C29—H29C	109.5
N4—C8—H8B	109.8	C28—C30—H30A	109.5
C7—C8—H8A	109.8	C28—C30—H30B	109.5
C7—C8—H8B	109.8	C28—C30—H30C	109.5
H8A—C8—H8B	108.2	H30A—C30—H30B	109.5
N3—C9—N4	124.5 (2)	H30A—C30—H30C	109.5
N3—C9—H9	117.8	H30B—C30—H30C	109.5
N4—C9—H9	117.8	C23—C31—H31	107.4
C11—C10—N4	118.8 (2)	C23—C31—C33	110.2 (3)
C11—C10—C15	122.7 (3)	C32—C31—C23	113.0 (3)
C15—C10—N4	118.5 (3)	C32—C31—H31	107.4
C10—C11—C19	123.7 (3)	C32—C31—C33	111.2 (3)
C12—C11—C10	116.9 (3)	C33—C31—H31	107.4
C12—C11—C19	119.4 (3)	C31—C32—H32A	109.5
C11—C12—H12	119.3	C31—C32—H32B	109.5
C13—C12—C11	121.4 (3)	C31—C32—H32C	109.5
C13—C12—H12	119.3	H32A—C32—H32B	109.5
C12—C13—H13	119.8	H32A—C32—H32C	109.5
C12—C13—C14	120.4 (3)	H32B—C32—H32C	109.5
C14—C13—H13	119.8	C31—C33—H33A	109.5
C13—C14—H14	119.3	C31—C33—H33B	109.5
C13—C14—C15	121.5 (3)	C31—C33—H33C	109.5
C15—C14—H14	119.3	H33A—C33—H33B	109.5
C10—C15—C16	123.4 (3)	H33A—C33—H33C	109.5
C14—C15—C10	117.1 (3)	H33B—C33—H33C	109.5
C14—C15—C16	119.5 (3)	O1—C34—H34A	109.2
C15—C16—H16	108.1	O1—C34—H34B	109.2
C15—C16—C17	110.5 (3)	O1—C34—C35	111.9 (4)
C15—C16—C18	112.0 (3)	H34A—C34—H34B	107.9
C17—C16—H16	108.1	C35—C34—H34A	109.2
C18—C16—H16	108.1	C35—C34—H34B	109.2
C18—C16—C17	110.0 (3)	C34—C35—H35A	109.5
C16—C17—H17A	109.5	C34—C35—H35B	109.5
C16—C17—H17B	109.5	C34—C35—H35C	109.5
C16—C17—H17C	109.5	H35A—C35—H35B	109.5
H17A—C17—H17B	109.5	H35A—C35—H35C	109.5
H17A—C17—H17C	109.5	H35B—C35—H35C	109.5
			
N1—C1—C2—C3	−55.0 (3)	C10—C11—C12—C13	−0.6 (4)
N1—C22—C23—C24	−179.0 (3)	C10—C11—C19—C20	123.8 (3)
N1—C22—C23—C31	2.3 (4)	C10—C11—C19—C21	−112.3 (3)
N1—C22—C27—C26	179.6 (3)	C10—C15—C16—C17	−111.7 (3)
N1—C22—C27—C28	−0.3 (4)	C10—C15—C16—C18	125.3 (3)
N3—C6—C7—C8	50.2 (3)	C11—C10—C15—C14	−0.9 (4)
N4—C10—C11—C12	179.7 (2)	C11—C10—C15—C16	178.6 (3)
N4—C10—C11—C19	−0.1 (4)	C11—C12—C13—C14	−0.5 (5)
N4—C10—C15—C14	−179.2 (3)	C12—C11—C19—C20	−56.0 (4)
N4—C10—C15—C16	0.2 (4)	C12—C11—C19—C21	67.9 (4)
C1—N1—C4—N2	−3.7 (4)	C12—C13—C14—C15	0.9 (5)
C1—N1—C22—C23	78.6 (3)	C13—C14—C15—C10	−0.3 (5)
C1—N1—C22—C27	−98.7 (3)	C13—C14—C15—C16	−179.7 (3)
C1—C2—C3—N2	49.4 (3)	C14—C15—C16—C17	67.7 (4)
C3—N2—C4—N1	−2.2 (4)	C14—C15—C16—C18	−55.3 (4)
C3—N2—C5—N3	71.5 (3)	C15—C10—C11—C12	1.3 (4)
C4—N1—C1—C2	32.8 (3)	C15—C10—C11—C19	−178.5 (3)
C4—N1—C22—C23	−107.3 (3)	C19—C11—C12—C13	179.2 (3)
C4—N1—C22—C27	75.4 (3)	C22—N1—C1—C2	−153.0 (2)
C4—N2—C3—C2	−21.6 (3)	C22—N1—C4—N2	−177.8 (2)
C4—N2—C5—N3	−107.3 (3)	C22—C23—C24—C25	−0.1 (5)
C5—N2—C3—C2	159.5 (2)	C22—C23—C31—C32	−137.6 (3)
C5—N2—C4—N1	176.6 (2)	C22—C23—C31—C33	97.3 (4)
C5—N3—C6—C7	161.4 (2)	C22—C27—C28—C29	124.2 (3)
C5—N3—C9—N4	174.5 (2)	C22—C27—C28—C30	−112.2 (3)
C6—N3—C5—N2	72.8 (3)	C23—C22—C27—C26	2.4 (4)
C6—N3—C9—N4	−1.0 (4)	C23—C22—C27—C28	−177.5 (3)
C6—C7—C8—N4	−55.2 (3)	C23—C24—C25—C26	1.3 (6)
C8—N4—C9—N3	−4.2 (4)	C24—C23—C31—C32	43.8 (4)
C8—N4—C10—C11	−99.0 (3)	C24—C23—C31—C33	−81.3 (4)
C8—N4—C10—C15	79.5 (3)	C24—C25—C26—C27	−0.6 (5)
C9—N3—C5—N2	−102.8 (3)	C25—C26—C27—C22	−1.2 (5)
C9—N3—C6—C7	−23.1 (4)	C25—C26—C27—C28	178.8 (3)
C9—N4—C8—C7	32.8 (3)	C26—C27—C28—C29	−55.7 (4)
C9—N4—C10—C11	75.0 (3)	C26—C27—C28—C30	67.9 (4)
C9—N4—C10—C15	−106.5 (3)	C27—C22—C23—C24	−1.8 (4)
C10—N4—C8—C7	−153.1 (2)	C27—C22—C23—C31	179.5 (3)
C10—N4—C9—N3	−178.2 (2)	C31—C23—C24—C25	178.6 (3)

### Hydrogen-bond geometry (Å, º)

**Table:**

*D*—H···*A*	*D*—H	H···*A*	*D*···*A*	*D*—H···*A*
O2—H2*A*···Br1	0.92 (2)	2.41 (3)	3.324 (4)	172 (10)
O2—H2*B*···Br1^i^	0.93 (2)	2.42 (2)	3.339 (4)	170 (5)
O1—H1···Br2	0.82	2.45	3.262 (3)	171
C3—H3*B*···Br1	0.97	2.83	3.660 (3)	144
C4—H4···Br2^ii^	0.93	2.85	3.733 (2)	158
C5—H5*A*···O1	0.97	2.50	3.383 (4)	151
C5—H5*B*···Br2^ii^	0.97	2.73	3.670 (3)	163
C6—H6*B*···Br1	0.97	2.89	3.741 (3)	147
C9—H9···O1	0.93	2.30	3.197 (4)	161

Symmetry codes: (i) −*x*, *y*+1/2, −*z*+3/2; (ii) −*x*, −*y*+1, −*z*+1.
